# Curcumin protection activities against γ-Rays-induced molecular and biochemical lesions

**DOI:** 10.1186/1756-0500-6-375

**Published:** 2013-09-21

**Authors:** Sameh S Tawfik, Amira M Abouelella, Yasser E Shahein

**Affiliations:** 1 Health Radiation Research Department, National Centre for Radiation Research and Technology (NCRRT), Atomic Energy Authority, P. O. Box; 29, Nasr City, Cairo, Egypt; 2 Radiation Biology Department, National Centre for Radiation Research and Technology (NCRRT), Atomic Energy Authority, P. O. Box; 29, Nasr City, Cairo, Egypt; 3 Molecular Biology Department, National Research Centre, P. O. Box: 33211, Dokki, Cairo, Egypt

**Keywords:** Curcumin, γ-rays, Biochemical alterations, DNA-profile, Mice

## Abstract

**Background:**

Curcumin is a yellow-pigment phenolic compound used as a food spice and has a broad spectrum of antioxidant, anti-carcinogenic, anti-mutagenic and anti-inflammatory properties.

**Methods:**

Radio-protective efficacy of curcumin; diferuloylmethane (C_21_H_20_O_6_) was evaluated using molecular and biochemical assays in male mice after exposure to 3 Gy γ-rays. Curcumin was given at a dose of 400 μmol/ kg body weight via gastric tubes for 5 following days either pre-, post- or both pre- and post-exposure.

**Results:**

The incidence of aberrant cells and aberration types (mostly chromatids, breaks and fragments) was reduced with curcumin dosage as compared to irradiated group. Thiobarbituric acid reactive substances (TBARS), hydroperoxide (HP), xanthine oxidase (XO) and apoptotic markers (DNA- fragmentation and caspase-3 activation) were increased significantly, whereas levels of glutathione (GSH) and the enzymatic antioxidants [Superoxide dismutase (SOD), catalase (CAT) and glutathione peroxidase (GPx)] were significantly depleted in γ-irradiated mice. Curcumin treatments of mice groups including the 5 days pre-irradiation treated group (protected), the 5 days post-irradiation treated group (treated), and the curcumin treated group 5 days pre- and post-irradiation (protracted), have attenuated the liver toxic effects of γ-rays as manifested by reducing the levels of TBARS, HP, XO and DNA fragmentation. Curcumin has also rescued the depletion of GSH and the enzymatic-antioxidant status.

**Conclusions:**

Curcumin has significant radio-protective and radio-recovery activities in γ-irradiated mice. It has antioxidant potential against γ-rays-induced cytogenetic, molecular and biochemical lesions in mice.

## Background

Ionizing radiation is known to induce oxidative stress through generation of reactive oxygen species (ROS) causing direct lesions in the DNA and biological molecules ultimately resulting in molecular and biochemical alterations [1]. *In vitro*, curcumin has a therapeutic potential for improving the antitumor effects of radiotherapy [2] and i*n vivo*, curcumin can modify cell survival and DNA repair efficacy [3].

Curcumin, a major bioactive compound present in turmeric is a yellow pigment phenolic compound obtained from the roots of *Curcuma longa* used as spice. It has a broad spectrum of antioxidant, anti-carcinogenic, anti-mutagenic and anti-inflammatory properties [2,4].

The anticancer potential of curcumin is attributed to its ability to treat various illnesses and suppress proliferation of a wide variety of tumour cells and down-regulate transcription factors [5]. Curcumin pre-treatment accelerated healing of irradiated wound and could be a substantial therapeutic strategy in the management of irradiated wounds [6].

Recently, Mosieniak et al. [7] found that curcumin-induced double-strand breaks promoting genetic instability by activating other cell signalling pathways and as a result, tumour cells fail to undergo cell cycle arrest, enter mitosis and die through mitotic catastrophe. In contrast, curcumin mitigates the genotoxic effects of the two well-known water contaminants arsenic and fluoride and ameliorated primary DNA damage in human peripheral blood lymphocytes [8]. Pretreatment with curcumin (737 mg/kg) protected against carbon tetra chloride (CCl_4_) toxicity of hepatic tissues. The attenuated hepatoprotection afforded by curcumin may be attributed to its low bioavailability *in vivo* (reduced absorption in the intestine and elevated intestinal metabolism). This postulation is supported by the findings that intra peritoneal injections of curcumin (368 mg/kg) induced GSH-antioxidant response and hepato protection to similar extents *in vivo*. The curcumin prooxidant can induce the GSH-antioxidant response and confer cytoprotection *in vitro*[9]. Human clinical trials indicated no dose-limiting toxicity for curcumin when administered at doses up to 10 g/day [10]. The current work describes the possible control measures against molecular and biochemical lesions in liver of whole body γ-irradiation in male mice and discusses the mechanism of action of curcumin.

## Methods

### Experimental animals

Male mice, 10–12 weeks, weighting (20 ± 2 g) were obtained from the Holding Company for Biological Products and Vaccines, Helwan, Egypt. Mice were kept under good ventilation and illumination conditions. Mice were allowed free access to a standard requirement diet and water *ad libitum*. The animals’ treatment protocol was approved by the ethical and scientific publishing committee of the National Centre for Radiation Research and Technology (NCRRT), Cairo, Egypt, following the guidelines of NIH.

### Radiation process

^137^Cs γ-irradiator (Gamma cell-40) was provided by the NCRRT, Cairo, Egypt, manufactured by the Atomic Energy of Canada. The dose rate was ~ 0.42 Gy/ min. Mice were placed in ventilated plastic cages and exposed to 3Gy γ-rays in groups of 6 mice simultaneously.

### Curcumin treatment

Curcumin was purchased from Sigma-Aldrich, USA. It was intra gastric given to mice on empty stomach, 3 h before feeding. The dose was 400 μmol/kg body wt/day based on protocol described by Thresiamma et al. [11]. The appropriate dose of curcumin per mouse was suspended in 0.5 ml of distilled water and given to the 3 mice groups as follows: 5 days pre-irradiation (protected group); 5 days post-irradiation (treated group); and both 5 days pre- and 5 days post-irradiation (protracted group) according to Okunieff et al. [12].

### Experimental design

Mice were divided into 6 groups treated as follows: Control group (n = 6): 0.5 ml of distilled water per mouse for 5 days. Curcumin group (n = 6): 0.5 ml of distilled water containing the appropriate dose of curcumin per mouse for 5 days. Irradiated group (n = 9): 0.5 ml of distilled water per mouse for 5 days prior to 3Gy γ-irradiation exposure. Protected group (n = 9): 0.5 ml of distilled water containing the appropriate dose of curcumin per mouse for 5 days prior to 3Gy γ-irradiation exposure. Treated group (n = 9): 0.5 ml of distilled water containing the appropriate dose of curcumin per mouse for 5 days post 3Gy γ-irradiation exposure. Protracted group (n = 9): 0.5 ml of distilled water containing the appropriate dose of curcumin per mouse both 5 days pre- and 5 days post 3Gy γ-irradiation exposure. Only six animals were sacrificed after 24 h from the last treatment or γ-rays exposure and liver tissue samples were excised. A high number of animals was used in the four γ-irradiated groups (n = 9), because of the elevated mortality rate that may occur in these groups. In the present experiment, only one mouse died in the γ-irradiated group.

### Cytogenetic technique

Colchicine (Sigma-Aldrich) was injected to mice via peritoneal cavity (0.3 ml/mouse of 0.025% colchicine in sterile deionized water) and animals were sacrificed by cervical dislocation 2 h later. Both femurs were dissected out and cleaned from the adhering tissue. Briefly, the bone marrow from femurs was aspirated and washed in saline, treated by hypotonic 0.56% KCl solution then, fixed in 3:1 methanol:glacial acetic acid. The metaphase plates were prepared by the routine air-drying method [13], dried and stained with 4% Giemsa. Chromosomal aberrations were scored under a light microscope. Chromatid and chromosome breaks, fragments, rings, dicentrics and polyploids were scored separately according to Bender et al. [14].

### Biochemical assays

Liver samples were quickly excised, washed with saline, blotted with a piece of filter paper and homogenized in appropriate buffer using a Branson sonifier (250, VWR Scientific, Danbury, Conn., USA). The homogenates were centrifuged at 800 × *g* for 5 min at 4°C to separate the nuclear debris. The supernatant so obtained was centrifuged at 10,500 × *g* for 20 min at 4°C to get the post-mitochondrial supernatant which was used to assay SOD activity.

In liver homogenates, the protein content was determined according to the method of Lowry et al. [15], using bovine serum albumin as standard. The activity of xanthine oxidase (XO) was assayed according to the method of Prajda and Weber [16]. The supernatant was pre-incubated for 40 min at 37°C and then added to the reaction mixture which contained in final concentrations: xanthine (0.17 μM); phosphate buffer (33 μM, pH 7.5); and a suitable amount of enzyme (supernatant). After centrifugation, xanthine oxidase activity was measured by the increase in absorbance at 293 nm. Total GSH was determined according to the methods of Ellman [17], which is based on the reduction of Ellman’s reagent [5,5-dithiobis-(2-nitrobenzoic acid)] by SH groups to form 1 mole of 2-nitro-5-mercaptobenzoic acid per mole of SH. The nitro-mercaptobenzoic acid has an intense yellow colour and can be determined spectrophotometrically at 412 nm. In details, protein precipitation was attained by mixing equal volumes of 10% aqueous homogenate and 7.5% sulfosalicylic acid followed by centrifugation at 600 × *g* for 15 min at 4°C. To 0.5 ml of the resulting supernatant, 2 ml of phosphate buffer (0.3 M, pH 7.7) and 0.25 ml of Ellman’s reagent (19.8 mg DTNB in 100 ml of 1% Na citrate) were added in a microcuvette and the absorbance was measured at 412 nm. SOD assay was based on the spectrophotometric assessment of the inhibition of nitro blue tetrazolium (NBT)-nicotinamide adenine dinucleotide (NADH) and phenazine methosulphate (PMS) mediated formazan formation by applying the technique of Kakkar et al. [18]. Absorbance was measured at 560 nm. One unit of SOD activity is defined, as the enzyme concentration required to inhibit chromogen production by 50% in one min/mg protein under the assay condition.

Estimation of lipid peroxidation (LP) indices as evidenced by the formation of TBARS and HP were measured in liver tissue by the method of Nichans and Samuelson [19] and Jiang et al. [20], respectively. In brief, 0.1 ml of tissue homogenate in Tris–HCl buffer, pH 7.5 was treated with 2 ml of (1:1:1 ratio) TBA-TCA-HCl reagent (Thiobarbituric acid 0.37%, 15% TCA, and 0.25 N HCl) and placed in water bath for 15 min, cooled and centrifuged at 1000 × *g* for 10 min. The absorbance of clear supernatant was measured against a reference blank at 535 nm. The results were expressed as LP μmol/mg protein. For hydroperoxide (HP) determination, 0.1 ml of homogenate was treated with 0.9 ml of Fox reagent (88 mg butylated hydeoxytoluene (BHT), 7.6 mg xylenol orange, and 9.8 mg ammonium ion sulphate were added to 90 ml of methanol and 10 ml 250 μM sulphuric acid) and incubated at 37°C for 30 min. The developed color was read at 560 nm.

CAT was assayed colorimetrically at 620 nm and expressed as Unite (μmol of H_2_O_2_ consumed/min) per mg protein as described by Sinha [21]. The reaction mixture (1.5 ml) contained 1.0 ml of 0.01 M phosphate buffer pH 7.0, 0.1 ml of tissue homogenate and 0.4 ml of 2 M H_2_O_2_. The reaction was stopped by adding 2.0 ml of dichromate-acetic reagent (5% potassium dichromate and glacial acid mixed in 1:3 ratio). Glutathione peroxidase (GPx) activity was assayed by method described by Ellman [17]. The assay mixture contained 0.2 ml of 0.4 M phosphate buffer, pH 7.0, 0.1 ml of 10 μM sodium azide, 0.2 ml of tissue homogenate (homogenized in 0.4 M phosphate buffer, pH 7.0), 0.2 ml glutathione, 0.1 ml of 0.2 mM H_2_O_2_. The reaction mixture was incubated at 37°C for 10 min. The reaction was stopped by adding 0.4 ml of 10% TCA, and centrifuged. Supernatant was measured for glutathione content by using Ellman’s reagent.

### DNA fragmentation

DNA was extracted and prepared according to the method described by Abou-Elella et al. [22]. In brief, liver tissues (100 mg) were treated with 100 mM Tris–HCl, 5 mM EDTA, 150 mM sodium chloride and 0.5% sarkosyl, pH 8.0, at 40°C for 10 min. Samples were incubated with ribonuclease (50 μg/ml) for 2 h at 37°C and proteinase K (100 μg/ml) at 48°C for 45 min. DNA was obtained by phenol:chloroform:isoamyl alcohol (25:24:1) (Sigma-Aldrich) extraction and precipitated with 0.3 M sodium chloride and cold isopropanol (1:1) at -20°C for 12 h. Cellular DNA was recovered by centrifugation of the sample at 20,800 × *g* at 4°C for 10 min. Thereafter, the precipitate was washed with 70% ethanol, dried and re-suspended in Tris buffer containing EDTA (10 mM Tris, 1 mM EDTA) at pH 8.0. Samples (100 μg DNA) were analyzed on a 1.5% agarose gel with ethidium bromide (0.5 μg/ml).

### Western blot

Immunoblot analysis was performed according to the method of Towbin et al. [23] with slight modifications using a NovaBlot semi-dry blotter (LKB, Bromma, Sweden). Briefly, 20 μg of the liver protein extracts from all groups were allowed to run on 12% SDS-PAGE after boiling with reducing SDS-PAGE loading buffer. Gels were either stained with Coomassie Brilliant Blue stain (R-250) to determine the molecular weight of the corresponding bands as manifested by the low molecular weight marker (GE healthcare, United Kingdom) or soaked in transfer buffer (16 mM Tris–HCl, 120 mM glycine and 20% methanol) prior to transfer to Immobilon-P transfer membrane (PolyVinylDimethylFluoride membrane, Millipore, Bedford, MA, USA). After transfer, membranes were incubated with blocking buffer (3% BSA in TBS, pH 7.5) for 1 h, washed three times with washing buffer (10 mM TBS containing 0.05% Tween 20) then incubated with gentle agitation overnight at room temperature with the polyclonal anti-caspase-3 (Santa Cruz, USA) in serum buffer (0.5% BSA in TBS containing 0.05% Tween 20, pH 7.5) at a dilution of 1:2000. Then, the antigen–antibody reaction was detected by incubating the membranes with anti-rabbit IgG peroxidase conjugate (Sigma–Aldrich, Saint Louis, MO, USA) at a dilution of 1:3000 in serum buffer for 1 h with gentle agitation at room temperature. The protein bands were visualized by incubating the membranes for 15–30 min in freshly prepared 4-chloro 1-naphthol (4C–1 N) developing solution (30 mg 4C-1 N) (MP Biomedicals, Inc., Fountain Pkwy, OH, USA) in 10 mM TBS containing 20% methanol and 0.06% H_2_O_2_. After color development, the membranes were washed twice with distilled water for about 30 min to stop the reaction, air-dried, and then photographed.

### Statistical analysis

Data were expressed as mean ± S.E of 6 mice. Statistical significance between 2 groups of parametric data was evaluated by one-way ANOVA method followed by Tukey’s post-test using the SPSS statistical package (SPSS 14.0 for Windows; SPSS, Inc, Chicago, IL). *P* < 0.05 was considered significant.

## Results

**Figure 1:**
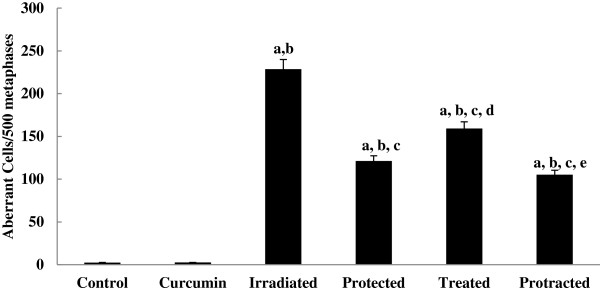
**Effect of curcumin on frequency of chromosomal aberrations of different mice groups.** All values are expressed as mean ± S.E., where (*n* = 6).^**a**^ Significant difference in comparing with control group. ^**b**^ Significant difference in comparing with curcumin group. ^**c**^ Significant difference in comparing with irradiated (3 Gy) group. ^**d**^ Significant difference in comparing with protected group. ^**e**^ Significant difference in comparing with treated group.

**Figure 2:**
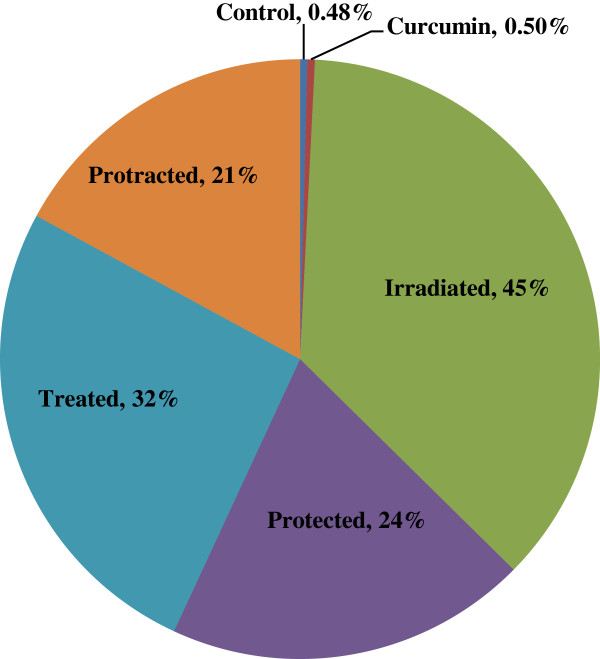
**Effect of curcumin on the percentage of aberrant cells as compared to normal cells in different mice groups.** All values are expressed as percentage of aberrant cells to normal cells, where (*n* = 6).

At time of sacrificing, only one mouse was died from irradiated group. Curcumin treated group produced a slight increase in the percentage of aberrant cells (0.5%), but it was not significantly different from the control group. γ-rays produced a significant increase in the percentage of aberrant metaphases and different types of aberrations compared to the control group. Aberrant cells percentage increased up to 45% at irradiated group (Figures 1 and 2).

The most common aberrations were breaks, fragments, rings and dicentrics (Table 1). Also, polyploid cells increased significantly above the control levels. Protected and treated groups with curcumin showed significant decreases, in the percentage of aberrant metaphases (24% and 32%), compared with irradiated group (45%) (Figure 2). In addition, curcumin treatment both 5 days pre- and 5 days post-γ-irradiation (protracted group) produced an additional significant decrease in percentage of aberrant cells, simply 21% (Figure 2), compared with irradiated group, since the repair was more effective than other groups when curcumin used as protracted treatment.

**Table 1:** Effect of curcumin on frequency of differential types of chromosomal aberrations of different mice groups

**Animal groups**	**Types of aberrations**/**500 metaphase**				
	**Chromatid breaks**	**Chromosome breaks**	**Fragments**	**Dicentrics** + **Rings**	**Polyploids**
**Control**	1.2 ± 0.3	0	2.0 ± 0.3	0	0
**Curcumin**	1.0 ± 0.5	0	2.1 ± 0.5	0	0
**Irradiated**	13.1 ± 0.2 ^**a,b**^	7.2 ± 0.2 ^**a,b**^	230 ± 8.7 ^**a,b**^	9.1 ± 0.2 ^**a,b**^	6.6 ± 0.5 ^**a,b**^
**Protected**	6.7 ± 0.5 ^**a,b,c**^	4.4 ± 0.6 ^**a,b,c**^	177.2 ± 7.9 ^**a,b,c**^	5.8 ± 0.4 ^**a,b,c**^	4.4 ± 0.6 ^**a,b,c**^
**Treated**	9.1 ± 0.2 ^**a,b,c,d**^	5.8 ± 0.4 ^**a,b,c,d**^	191.3 ± 9.7 ^**a,b,c**^	7.2 ± 0,2 ^**a,b,c,d**^	4.8 ± 0.6 ^**a,b,c**^
**Protracted**	5.0 ± 0.5 ^**a,b,c,d,e**^	4.3 ± 0.4 ^**a,b,c,e**^	158.2 ± 5.5 ^**a,b,c,d,e**^	4.9 ± 0.9 ^**a,b,c,d,e**^	4.3 ± 0.3 ^**a,b,e**^

All values are expressed as mean ± S.E., where (*n* = 6).

^**a**^ significant difference in comparing with control group.

^**b**^ significant difference in comparing with curcumin group.

^**c**^ significant difference in comparing with irradiated (3 Gy) group.

^**d**^ significant difference in comparing with protected group.

^**e**^ significant difference in comparing with treated group.

All protected, treated and protracted mice groups had significant increases in chromosomal aberration frequency, compared with control group, because the repairing efficacy of the curcumin models of treatments did not reverse the induced aberrations to the control level (Figures 1 and 2).

Qualitatively, the three curcumin models of treatments significantly reduced the frequency of the major aberrations like breaks and fragments. The protracted protocol of treatment was more effective and significantly lowered all types of aberrations as well as polyploids (Table 1).

**Figure 3:**
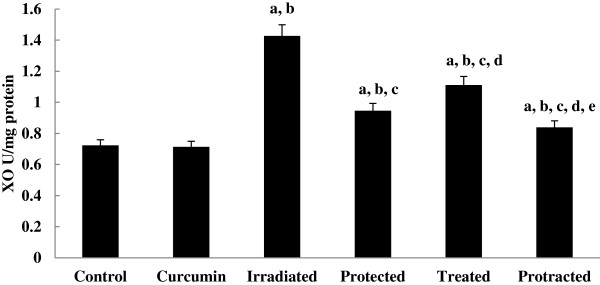
**Effect of curcumin on pro-oxidant enzyme**; **xanthine oxidase (XO) in liver tissue of different mice groups.** All values are expressed as mean ± S.E., where (*n* = 6). ^**a**^ Significant difference in comparing with control group. ^**b**^ Significant difference in comparing with curcumin group. ^**c**^ Significant difference in comparing with irradiated (3 Gy) group. ^**d**^ Significant difference in comparing with protected group. ^**e**^ Significant difference in comparing with treated group.

Xanthine oxidase (XO) activity was increased significantly in irradiated group, which was decreased significantly with curcumin-treatment either pre-, post- or both pre- and post-γ-irradiation. However, protracted treatment decreased XO activity more significantly compared to both protected and treated groups (Figure 3).

**Figure 4:**
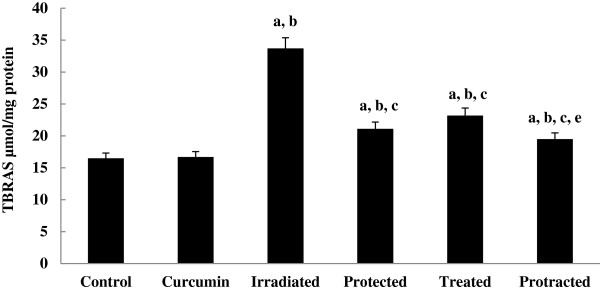
**Effect of curcumin on level of TBARS in liver tissue of different mice groups.** All values are expressed as mean ± S.E., where (*n* = 6).^**a**^ Significant difference in comparing with control group. ^**b**^ Significant difference in comparing with curcumin group. ^**c**^ Significant difference in comparing with irradiated (3 Gy) group. ^**d**^ Significant difference in comparing with protected group. ^**e**^ Significant difference in comparing with treated group.

**Figure 5:**
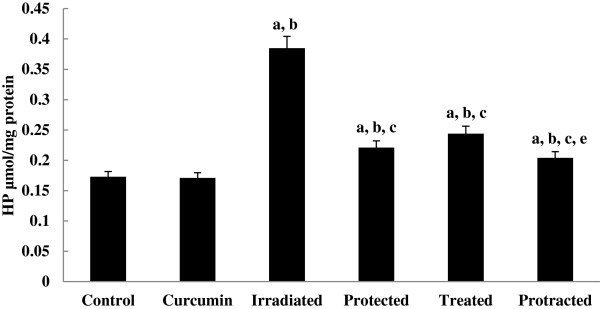
**Effect of curcumin on level of hydroperoxide (HP) in liver tissue of different mice groups.** All values are expressed as mean ± S.E., where (*n* = 6). ^**a**^ Significant difference in comparing with control group. ^**b**^ Significant difference in comparing with curcumin group. ^**c**^ Significant difference in comparing with irradiated (3 Gy) group. ^**d**^ Significant difference in comparing with protected group. ^**e**^ Significant difference in comparing with treated group.

The levels of TBARS and HP (Figures 4 and 5) were increased significantly in irradiated group, which were decreased significantly on treatment with curcumin either pre-, post- or both pre- and post-γ-irradiation. The decrease was more significant in protracted group compared to both protected and treated groups.

**Figure 6:**
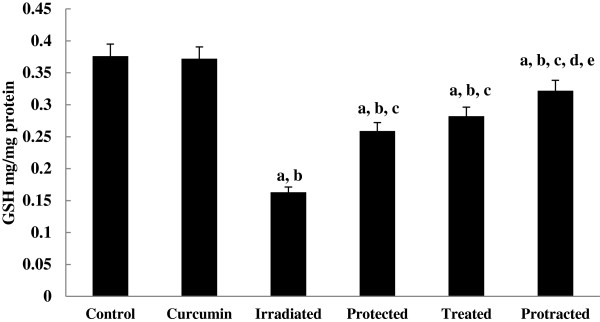
**Effect of curcumin on level of GSH in liver tissue of different mice groups.** All values are expressed as mean ± S.E., where (*n* = 6). ^**a**^ Significant difference in comparing with control group. ^**b**^ Significant difference in comparing with curcumin group. ^**c**^ Significant difference in comparing with irradiated (3 Gy) group. ^**d**^ Significant difference in comparing with protected group. ^**e**^ Significant difference in comparing with treated group.

**Figure 7:**
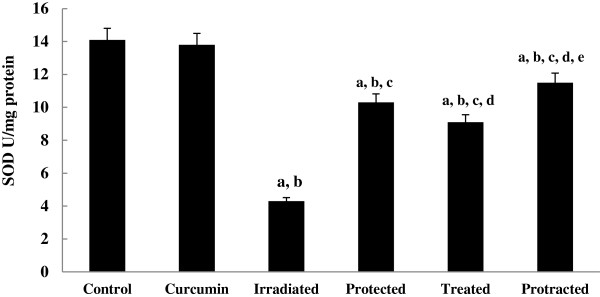
**Effect of curcumin on SOD activity in liver tissue of different mice groups.** All values are expressed as mean ± S.E., where (*n* = 6). ^**a**^ Significant difference in comparing with control group. ^**b**^ Significant difference in comparing with curcumin group. ^**c**^ Significant difference in comparing with irradiated (3 Gy) group. ^**d**^ Significant difference in comparing with protected group. ^**e**^ Significant difference in comparing with treated group.

**Figure 8:**
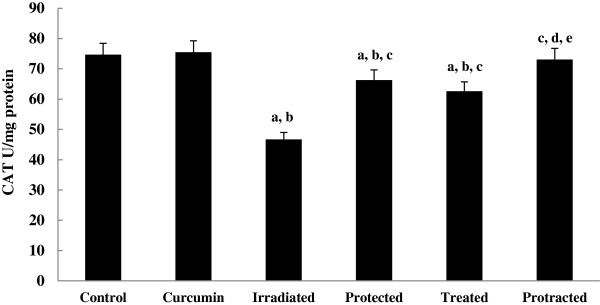
**Effect of curcumin on CAT activity in liver tissue of different mice groups.** All values are expressed as mean ± S.E., where (*n* = 6).^**a**^ Significant difference in comparing with control group. ^**b**^ Significant difference in comparing with curcumin group. ^**c**^ Significant difference in comparing with irradiated (3 Gy) group. ^**d**^ Significant difference in comparing with protected group. ^**e**^ Significant difference in comparing with treated group.

**Figure 9:**
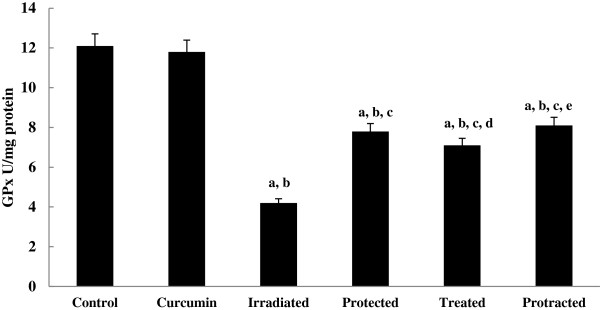
**Effect of curcumin on GPx activity in liver tissue of different mice groups.** All values are expressed as mean ± S.E., where (*n* = 6). ^**a**^ Significant difference in comparing with control group. ^**b**^ Significant difference in comparing with curcumin group. ^**c**^ Significant difference in comparing with irradiated (3 Gy) group. ^**d**^ Significant difference in comparing with protected group. ^**e**^ Significant difference in comparing with treated group.

The levels of non-enzymatic antioxidant; GSH (Figure 6) and enzymatic antioxidants; SOD, CAT and GPx (Figures 7, 8 and 9) were significantly depleted in irradiated group, which were increased significantly on treatment with curcumin either pre-, post- or both pre- and post-γ-irradiation. The protracted treatment was found to be more effective compared to both protected and treated groups.

**Figure 10:**
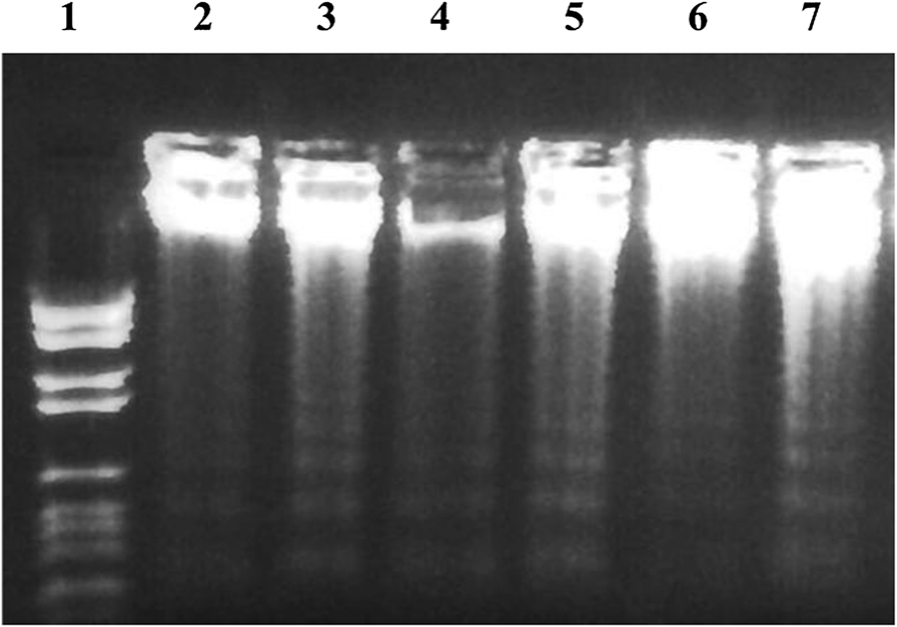
**Effect of curcumin on DNA fragmentation in mouse liver cells.** Lane 1 represents: DNA molecular weight marker, lane 2: control group, lane 3: curcumin group, lane 4: irradiated group, lane 5: protected group, lane 6: treated group and lane 7: protracted group.

**Figure 11:**
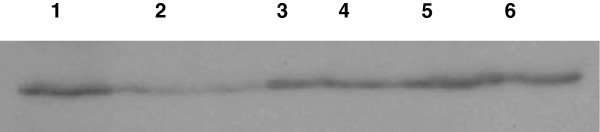
**Caspase**-**3 expression by western blot.** Lane 1: control group, lane 2: irradiated group, lane 3: curcumin group, lane 4: protected group, lane 5: treated group and lane 6: protracted group.

The administration of curcumin pre-γ-exposure reduced apoptosis as measured by DNA-fragmentation (Figure 10, Lane 5). In current experiments, the DNA fragmentation in the mouse liver cells was also recovered by the administration of curcumin to treated and protracted groups (Figure 10, Lane 6 and 7). Caspase-3 cleavage was not affected in all groups except the gamma irradiated group (Figure 11, Lane 2).

## Discussion

A dose-dependent spectrum of radiation-induced chromosome aberrations such as dicentrics, translocation and centric ring was recorded for effective radiation dose [24]. Radiation causes a large spectrum of DNA lesions and the ability to activate repairer pathway is essential to maintain genomic stability and restore normal function [25]. Curcumin could be used as a radio protective agent due to its ability to reduce oxidative stress and inhibit transcription of genes related to oxidative stress and inflammatory responses [26]. Curcumin, as a non-genotoxic agent reduced the DNA damage, retarded ROS generation and LP and raised the level of antioxidant activity [27].

In the present study, curcumin at testing dose and duration, alone did not significantly induced aberrations, confirming its non-mutagenicity. Augmentation in chromosomal aberrations was reported in the bone marrow of irradiated mice [28], which is proved by the present data. Chromosomal aberration frequency increased significantly in irradiated group, which were decreased significantly on treatment with curcumin either pre-, post- or both pre- and post-γ-irradiation. However, protracted treatment decreased their frequency more significantly compared to both protected and treated groups. These results indicates that the antioxidant curcumin possess both protection and repair properties against chromosome damage produced by radiation. Thresiamma et al. [11] found that curcumin significantly reduces the number of bone marrow cells with chromosomal aberrations and chromosomal fragments as effectively as alpha-tocopherol. Moreover, curcumin possess therapeutic properties to scavenge free radicals and to inhibit clastogenesis in human cells. Furthermore, Alaikov et al. [29] indicated that curcumin has pleiotropic effects on signal transduction by inhibiting transcription. Curcumin modifies signal transduction pathways, inflammatory cytokines and enzymes and gene products linked with cell survival [30].

Data revealed that, pro-oxidant enzyme, lipid peroxidative indices and the non-enzymatic- and the enzymatic-antioxidants levels do not differ from control levels in mice group treated with curcumin alone.

Curcumin is known to protect bio membranes against per-oxidative damage. Peroxidation of lipids is known to be a free radical-mediated chain reaction leading to the damage of the cell membrane [31]. Moreover, curcumin belongs to the family of polyphenolic compounds which modulate the activities of the pro-inflammatory enzymes via regulation of the antioxidant response elements [4]. Furthermore, it has protective effects against hepatic ischemia/reperfusion injury. Its mechanism might be related to the over expression of heat shock protein and antioxidant enzymes [32]. Additionally in the present study, whole body γ-exposure of mice to 3 Gy has induced significant increases in XO activity. Most of the toxic effects of ionizing radiation to normal tissue are due to the generation of ROS which triggers formation of several reactive intermediates [33]. To overcome such events, living cells are equipped with integrated endogenous enzymatic and antioxidant systems such as SOD, CAT, GPx and GSH [34]. Free radicals generated by irradiation also react with poly unsaturated fatty acid (PUSFAs) generating HP, which in turn can induce changes in the lipid bilayer thereby altering the membrane permeability and inducing LP [27].

Zhang et al. [35] concluded that ROS generated by γ-radiation induced membrane LP and cellular DNA-damage. In the present study, curcumin reduced the LP content of the liver tissue sufficiently. This may ascribe to the induction of antioxidant enzyme activities by curcumin, which consequently mitigate the cell membrane LP damage. In the present study, the levels of LP and HP, the end-products of LP are significantly increased in liver tissue of irradiated group. These results are in agreement with a recent study of Sinha et al. [36]. Also, curcumin treatment significantly decreased LP and HP levels in various tissues which were accordance with Wang et al. [37]. The authors suggest that the anti-lipoperoxidative effect of curcumin may be explained by its direct free radical scavenger property.

Superoxide dismutase and catalase enzymes are present in many animal cells [38]. SOD is an oxygen radical scavenger that converts superoxide anion radicals to HP and protects living cells against damage. CAT is an oxidoreductase that catalyses the conversion of HP to water and oxygen, also can protect living cells from damage induced by ischemia/reperfusion through scavenging ROS. A recent study had shown that dietary curcumin could increase antioxidant enzyme expression and activity in tissue, inhibit ROS, protect cell function from oxidative stresses and improves survival in mice [39]. The results of the present study showed that treatment with curcumin could increase SOD, CAT and GPx-activities and decrease TBARS and HP concentrations, suggesting that curcumin treatment also increases antioxidative bioactive molecule expression in liver after irradiation injury and attenuates ROS damage in liver.

Apoptosis is a fundamental process essential for both development and maintenance of tissue homeostasis. Cells undergoing apoptosis exhibit specific changes including chromatin condensation, DNA fragmentation, caspases activity and nuclear breakdown [40]. Curcumin reduces active caspase-3 and DNA-fragmentation which were induced by γ-radiation by attenuating relating signalling pathways [35].

The identification of caspase-3 activity modifications during the cell death induced by γ-rays in liver cells can help in the insight of the causal molecular mechanisms responsible for the induction of apoptosis and necrosis cell death pathways [41]. We showed that the administration of curcumin also reduced the effects of γ-rays on DNA fragmentation, while the caspase-3 cleavage was not statistically affected in all groups, except the irradiated group. In contrast, Abouelella et al. [42] found that intake of *Echinacea purpurea* was not effective to reduce the apoptotic mechanisms induced by gamma-rays in mouse liver. In fact, curcumin was able to slightly enhance DNA fragmentation in all groups. Nevertheless, more studies are needed in order to confirm these findings.

## Conclusions

These observations show that curcumin exerts its protective effect by decreasing the LP and improving antioxidant status. These results may provide the molecular basis for the application of curcumin in clinical radiation therapy.

## Competing interests

The authors declare that they have no competing interests.

## Authors’ contributions

ST, AA, and YS designed the study, undertook all experimental work, drafted the initial manuscript and reviewed the manuscript for important intellectual content and read and approved the final version.
